# Acupuncture Extended the Thrombolysis Window by Suppressing Blood–Brain Barrier Disruption and Regulating Autophagy–Apoptosis Balance after Ischemic Stroke

**DOI:** 10.3390/brainsci14040399

**Published:** 2024-04-19

**Authors:** Zhihui Zhang, Tianliang Lu, Shanshan Li, Ruyu Zhao, Honglei Li, Xinchang Zhang, Yiyang Li, Yawen Xia, Guangxia Ni

**Affiliations:** 1 College of Acupuncture-Moxibustion and Tuina, Nanjing University of Chinese Medicine, No. 138 Xianlin Avenue, Qixia District, Nanjing 210023, China; zhihuizhang@njucm.edu.cn (Z.Z.);; 2 Key Laboratory of Acupuncture and Medicine Research of Ministry of Education, Nanjing University of Chinese Medicine, No. 138 Xianlin Avenue, Qixia District, Nanjing 210023, China

**Keywords:** acupuncture, rt-PA thrombolysis, ischemic stroke, blood–brain barrier, apoptosis

## Abstract

Background: Ischemic stroke (IS) is one of the leading causes of death and disability worldwide. The narrow therapeutic window (within 4.5 h) and severe hemorrhagic potential limits therapeutic efficacy of recombinant tissue type plasminogen activator (rt-PA) intravenous thrombolysis for patients. Xingnao Kaiqiao (XNKQ) acupuncture is an integral part of traditional Chinese medicine, specifically designed to address acute ischemic stroke by targeting key acupoints such as Shuigou (GV26) and Neiguan (PC6). In this study, we explored the therapeutic potential of XNKQ acupuncture in extending the time window for thrombolysis and interrogated the molecular mechanisms responsible for this effect. Methods: The effect of extending the thrombolysis window by acupuncture was evaluated via TTC staining, neuronal score evaluation, hemorrhagic transformation assay, and H&E staining. RNA sequencing (RNA-seq) technology was performed to identify the therapeutic targets and intervention mechanisms of acupuncture. Evans blue staining and transmission electron microscopy were used to assess blood–brain barrier (BBB) integrity. Immunofluorescence staining and co-immunoprecipitation were performed to evaluate the level of autophagy and apoptosis and validate their interactions with BBB endothelial cells. Results: Acupuncture alleviated infarction and neurological deficits and extended the thrombolysis window to 6 h. The RNA-seq revealed 16 potential therapeutic predictors for acupuncture intervention, which related to suppressing inflammation and restoring the function of BBB and blood vessels. Furthermore, acupuncture suppressed BBB leakage and preserved tight junction protein expression. The protective effect was associated with regulation of the autophagy–apoptosis balance in BBB endothelial cells. Acupuncture intervention dissociated the Beclin1/Bcl-2 complex, thereby promoting autophagy and reducing apoptosis. Conclusion: XNKQ acupuncture could serve as an adjunctive therapy for rt-PA thrombolysis, aiming to extend the therapeutic time window and mitigate ischemia–reperfusion injury. Acupuncture suppressed BBB disruption by regulating the autophagy–apoptosis balance, which in turn extended the therapeutic window of rt-PA in IS. These findings provide a rationale for further exploration of acupuncture as a complementary candidate co-administered with rt-PA.

## 1. Introduction

Ischemic stroke (IS) is a major cause of death and disability in China, accounting for 86.8% of strokes [1]. Ischemic stroke is characterized by a sudden reduction in cerebral blood flow, resulting in damage to brain cells [2]. Within 4.5 h of the onset of IS, the administration of recombinant tissue type plasminogen activator (rt-PA) through intravenous thrombolysis is widely regarded as the most effective treatment, as it helps restore neurological function and enhance prognosis [3]. However, due to the limited therapeutic time window and the occurrence of hemorrhagic transformation, only a minority of patients receive this treatment in time and fully benefit from it [4,5,6]. New therapies are urgently needed to reduce side effects and extend the thrombolysis window.

Blood–brain barrier (BBB) disruption is considered one of the pathophysiological characteristics of IS, which promotes the progression of vasogenic edema formation and hemorrhagic transformation [7]. Maintaining BBB integrity is instrumental in attenuating ischemia–reperfusion injury. The BBB is composed of brain microvascular endothelial cells (BMECs), astrocytes, pericytes, neurons, and the surrounding extracellular matrix of the vasculature [8]. As a unique metabolic and immune barrier, the BBB selectively regulates the transport of molecules, and protects the brain from potentially harmful substances and inflammatory factors in the blood. The vascular endothelium, which acts as the primary structural and functional component of the cerebral microvasculature, plays a crucial role in preserving its regular physiological function [9]. BMECs are the scaffolding of the BBB; the demise of BMECs compromises the BBB’s integrity. Apoptosis is the predominant form of cellular demise following stroke, mediating vascular damage and neuronal injury. When stroke occurs, ischemia-induced brain endothelial injury, coupled with endothelial inflammation and subsequent functional impairment, results in increased vascular permeability and BBB leakage, culminating in ischemic brain injury [10,11]. In addition, delayed treatment with rt-PA leads to hyperperfusion, resulting in extracellular matrix degradation and BMECs death, as well as further BBB disruption [12]. Therefore, identifying the mechanisms in restoring BBB integrity and comprehending the mediators in BMECs dysfunction may be an effective therapeutic method for the treatment of IS.

Acupuncture, as an important element of traditional Chinese medical technique, has been used for stroke treatment for centuries [13]. Xingnao Kaiqiao (XNKQ) acupuncture, developed by Professor Xuemin Shi, has shown promising results in the management of stroke, specifically in treating acute ischemic stroke [14]. Shuigou (GV26) and Neiguan (PC6) are the most significant acupoints of XNKQ acupuncture. GV26 was considered an important point to arouse the brain in emergency situations by ancient doctors, due to its ability to increase blood flow in the carotid artery and improve cerebral circulation. PC6 is the Luo connecting point of the pericardium channel, which improves the cardiac output of stroke patients and improves the supply of oxygen to the brain. Evidence-based medical research has confirmed that acupuncture improves the cerebral blood flow status in the ischemia area of brain and significantly relieves stroke symptoms [15,16,17]. In addition, acupuncture also has significant therapeutic effects in traumatic brain injury [13]. Recently, several clinical studies have found that XNKQ acupuncture can be used as an adjuvant therapy to enhance the efficacy of rt-PA thrombolysis within 4.5 h [14,18,19]. Simultaneous treatment with XNKQ acupuncture and rt-PA improve the safety of thrombolytic therapy, reducing the risk of hemorrhagic transformation caused by rt-PA administration. The available evidence indicates that XNKQ acupuncture has the potential to reduce complications of thrombolytic therapy. However, no study to date has explored the therapeutic potential of acupuncture in extending the time window for rt-PA thrombolysis, and the mechanism behind this process has also yet to be elucidated.

RNA sequencing (RNA-seq) is a pivotal genomic technique characterized by high throughput and ease of quantification. It enables detailed analysis of gene expression, which is crucial for understanding cellular processes and disease mechanisms [20]. This advanced technique allows researchers to meticulously analyze and quantify gene expression changes that occur during the ischemia and reperfusion phase, pinpointing the critical targets that contribute to the disease’s pathogenesis [21]. By identifying these indicators, RNA-seq not only aids in unraveling the molecular underpinnings of ischemia–reperfusion injury, but also opens avenues for the development of targeted therapies, potentially revolutionizing personalized treatment approaches to mitigate the impact of this prevalent clinical challenge [22].

In this study, we confirmed the role of XNKQ acupuncture on the thrombolysis time window extension and ischemia–reperfusion injury alleviation. Based on RNA-seq technology, we characterized gene expression profiles to identify the therapeutic targets and intervention mechanisms of acupuncture after delayed rt-PA thrombolysis. More importantly, we figured out the underlying mechanism of acupuncture on BBB integrity through assessing the effect on apoptosis and autophagy.

## 2. Materials and Methods

### 2.1. Animals

Adult male Sprague Dawley (SD) rats weighing 320 ± 20 g at SPF level were supplied by Beijing Weitonglihua Animal Co., Ltd. (Beijing, China; license no. SCXK (Jing): 2021-0011.) Every rat was kept in a room with controlled temperature and humidity, following a 12 h light/dark cycle. The Institutional Animal Care and Use Committee of Nanjing University of Chinese Medicine (202309A048) granted approval for the animal experiments conducted in this study, which adhered strictly to the guidelines set by the National Institutes of Health Animal Care and Use Committee.

### 2.2. Animal Grouping

The rats were divided into different groups, including Sham, Model, rt-PA, and rt-PA+Acu using a random method. In addition, rats in the rt-PA and rt-PA+Acu groups were further divided into three subgroups according to the time of administration of rt-PA intravenous thrombolysis (4.5 h, 6 h, 7.5 h). Specifically, the Model group represents the ischemic stroke condition without receiving either thrombolytic therapy with rt-PA or acupuncture, which is essential for establishing a baseline of the disease state. In addition, the Sham group was designed to eliminate the influence of non-pathological factors from the surgical procedure and was not subject to injection of a thromboembolus into the middle cerebral artery after the carotid artery dissection.

### 2.3. The Embolic Stroke Model Establishment

According to the protocol described by Zhang et al., a blood clot was inserted into the middle cerebral artery (MCA) to induce an embolic stroke model [23]. Firstly, to prepare the embolus, the donor rat’s external carotid artery (ECA) was catheterized, and blood was collected in PE-50 tubing from aorta ventralis. After clotting for 2 h at 37 °C and refrigerating for 22 h, a clot-filled segment of the tube was transferred to a PE-10 tube for aspiration and rinsing of the clot to remove red blood cells. Then, a modified PE-50 catheter connected to a syringe was used to inject the clot into the MCA. Next, anesthetized rats were fixed and then subjected to skin disinfection. After the cervical blood vessels were exposed, ligation and temporary clamping were applied to the ECA, while a partial arteriotomy was performed. The modified PE-50 tube containing the blood clot was introduced into the ECA and progressed towards the ICA until it reached the origin of the MCA. Following a minor withdrawal, the clot was gradually infused with saline solution. The catheter was removed after 5 min. The model’s success was confirmed based on cerebral blood flow obstruction. The laser speckle imaging system (RWD, Shenzhen, China) was utilized to monitor cerebral blood flow, and the presence of successful obstruction was determined via a significant reduction in perfusion (Figure S1A,B).

### 2.4. Acupuncture Treatment

**Figure 1:**
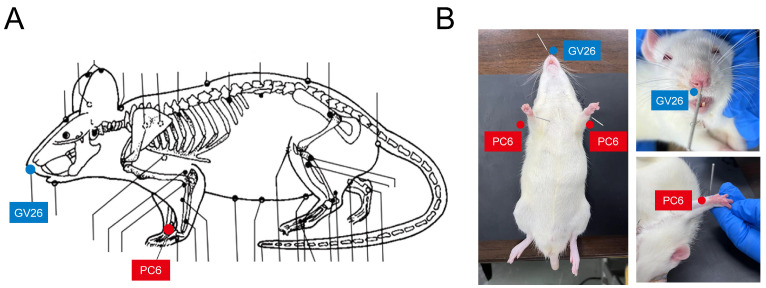
Schematic of Xingnao Kaiqiao acupuncture. (**A**) The locations of the acupoints in rats. Shuigou (GV26, in blue) and bilateral Neiguan (PC6, in red) acupoints are labeled. (**B**) Schematic images of an experimental rat, showing the location of acupoints GV26 and PC6.

After the model was established, rats in the acupuncture group were treated with acupuncture at the Shuigou (GV26) and bilateral Neiguan (PC6) acupoints at 2 h post model establishment. For the acupuncture, we used stainless steel disposable acupuncture needles, sized 0.3 mm × 13 mm. Acupoints were identified using the acupoint map of experimental animals issued by the Chinese Acupuncture Society (Figure 1).

The location of PC6 was about 3 mm proximal to the palm crease, positioned above the median nerve. Bilateral PC6 points were stimulated by inserting 3 mm vertically and applying a reducing technique with light insertion and heavy lifting, combined with twirling motions (anticlockwise with the left hand and clockwise with the right, amplitude under 90 degrees, and a frequency of 120–160 twists per minute) for 1 min. The GV26 acupoint was located at the junction of the upper 1/3 and middle 1/3 of the upper lip. After given acupuncture at PC6, the GV26 acupoint was punctured obliquely to a depth of 2–3 mm and treated with sparrow-pecking needling until the eye became wet (about 1 min). The needles were retained, and the whole treatment duration of acupuncture was 30 min.

### 2.5. rt-PA Thrombolysis Treatment

Rats in the rt-PA and rt-PA+Acu groups were injected with 10 mg/kg rt-PA through the tail vein at the indicated times after model establishment.

### 2.6. Behavioral Testing

After surgery for 24 h, neurological function was scored using the modified Berderson et al. clinical scale: 0, no apparent neurological deficit; 1, contralateral forelimb flexion; 2, decreased grip of the contralateral forelimb while tail pulled; 3, spontaneous movement in all directions and contralateral circling only if pulled by tail; 4, spontaneous contralateral circling [24]. The Corner test was used to evaluate limb coordination function. This test needs a special device formed by two plates at a 30° angle. When the rat reaches the corner, the whiskers are stimulated by the borders on both sides, leading the rat to turn left or right. Healthy rats typically show approximately equal numbers of left and right turns, but rats with unilateral cerebral ischemic injury preferentially turn towards the affected side. The test was conducted 24 h after modeling, repeated ten times with a 30-s interval each time, and the final record was the ratio of pathological turns to the total number of turns.

### 2.7. Measurement of Infarct Volume

The brain tissues were rapidly extracted from the anesthetized rats 24 h after successful modelling and cut into 2 mm sections using brain matrices (RWD, Shenzhen, China). Subsequently, the brain slices were placed in a 2% TTC solution (Sigma-Aldrich, St. Louis, MO, USA) at 37 °C for 15 min and then fixed in 4% formaldehyde solution for a period of 24 h. Finally, the scanned images of the brain slices were analyzed using ImageJ software (version 1.5.4). The regions of brain tissues that were not stained were identified as the areas affected by infarction. These areas can be calculated using the following formula: infarcted brain volume (%) = (total brain volume − stained brain volume)/total brain volume × 100%.

### 2.8. Measurement of Hemorrhagic Transformation

Hemorrhagic transformation was assessed by measuring the levels of hemoglobin in the ischemic hemisphere using a spectrophotometric assay as previously described [25]. Rats were sacrificed via transcranial perfusion 24 h after the onset of ischemia, and the tissue of the ischemic hemisphere was dissected and separated. Brain tissues were homogenized with 2 mL PBS. The homogenate was then centrifuged at 13,000 rpm for 30 min, and the supernatants were collected. Finally, hemoglobin levels were measured using a hemoglobin assay kit (QuantiChrom^TM^, Holland, OH, USA). A microplate reader was used to measure the optical density value.

### 2.9. Hematoxylin and Eosin Staining

Following a 24-h period of cerebral infarction, anesthetized rats underwent transcardial perfusion by using pre-chilled saline and 4% paraformaldehyde. The brain tissues were rapidly removed and then fixed in a 10% formalin solution for 24–48 h. After dehydration of the brain tissues, they were embedded in paraffin. The paraffin tissues were sectioned into 6-μm thick slices using microtomy, placed on glass slides, and dried at 80 °C for 2 h. The deparaffinized sections underwent staining [26]. After the process of staining, the tissue sections were mounted with yellow-colored pinene resins and observed for histological changes in brain tissue under a light microscope (Olympus, Tokyo, Japan) at 100× and 400× magnifications.

### 2.10. TUNEL Staining

Brain tissue sections were treated using the TUNEL staining kit (Elabscience, Wuhan, China) to label DNA fragments with fluorescein-dUTP and detect the number of apoptotic neurons. Cell nuclei were stained in blue, while positive nuclei were stained in green. Randomly selecting 5 fields of view under a fluorescence microscope, the number of positively labeled nuclei was manually counted to determine the number of positive nuclei. The percentage of apoptotic cells was calculated as the ratio of TUNEL-positive cells to the total cell count.

### 2.11. Measurement of Blood–Brain Barrier Permeability

To examine Blood–Brain Barrier (BBB) permeability following embolic stroke, stroked rats were injected with 2% Evans blue (EB) dye (Sigma-Aldrich, St. Louis, MO, USA) at a dose of 0.4 mL/100 g via the tail vein 2 h before sacrifice. After anesthesia, the rats underwent myocardial perfusion with cooled saline. Brain tissues were rapidly removed and then separated into hemispheres. The right hemisphere was weighed and homogenized in 500 μL of formamide per 100 mg tissue. The homogenate was then incubated in a water bath at 60 °C for 24 h, followed by centrifugation at 10,000× *g* for 20 min at 4 °C. The absorbance of EB in the supernatant was measured at 620 nm using a spectrophotometer. The content of EB (μg/g) was calculated using the formula: EB concentration (μg/mL) × volume of formamide (mL)/mass of brain tissue (g). This method was based on the content of EB in brain tissue to observe the changes in BBB permeability.

BBB permeability was also assessed using frozen sections. After cardiac perfusion, fixation and dehydration, brain tissues were embedded in OCT and then sliced in 25 μm frozen sections. The expression of EB fluorescence in the coronal brain sections was visualized using a fluorescence microscope (Leica Thunder, Wetzlar, Germany).

### 2.12. Library Preparation, RNA Sequencing and Bioinformatic Analysis

Total RNA was isolated and purified using a TRIzol reagent (Invitrogen, Waltham, MA, USA). The Bioanalyzer 2100 (Agilent, Waldbronn, Germany) was used to evaluate the RNA integrity, ensuring a RIN number higher than 7.0. We conducted paired-end sequencing (PE150) using an Illumina Novaseq TM 6000 (Illumina, San Diego, CA, USA). Following the demultiplexing process, the reads were aligned to the rat reference genome (mRatBN7.2) using the STAR alignment tool (version 2.7.10). Salmon (version 1.9.0) was used to count uniquely mapped read pairs using the Ensembl rat gene annotation to create a raw read count table.

The R package DESeq2 (version 1.36) [27] was utilized to conduct differential expression analyses on count data. Differentially expressed genes were determined by setting the threshold at |log2FC| > 1 and adjusted *p* < 0.05. We utilized the clusterProfiler package (version 3.14) to perform over-representation analysis in order to identify significant Gene Ontology (GO) Biological and Kyoto Encyclopedia of Genes and Genomes (KEGG) Pathways [28]. In addition, the plasma miRNAs expression data between ischemic stroke patients with or without rt-PA treatment (GSE95204) were also acquired for further identification of predictors of acupuncture treatment. The MiRDB database was used for thrombolytic-related miRNA target prediction [29].

### 2.13. Transmission Electron Microscopy

Rats that had been anesthetized were firstly perfused with PBS, followed by perfusion with 2.5% glutaraldehyde through the heart. The brain tissue was rapidly collected and sliced into 1 mm^3^ tissue blocks, then immersed in 2.5% glutaraldehyde solution at 4 °C overnight. After being washed with PBS three times, these tissue blocks were then fixed with 1% osmium tetroxide solution for 2 h and washed again with PBS three times. The tissues were dehydrated in gradient alcohol twice for 20 min each. After being treated with pure acetone for 20 min, the tissues were embedded and incubated at 70 °C overnight. Ultrathin sections of 80 nm thickness were cut using a Reichert ultramicrotome. Lastly, the tissue sections were stained with 3% uranyl acetate and lead citrate. TEM was used to determine the ultrastructural changes of BBB.

### 2.14. Western Blotting

Total proteins were extracted from the right cerebral cortex in the ischemic penumbra and quantitated using a BCA kit (Beyotime, Shanghai, China). Equal amounts of protein were separated with 8–12% SDS-PAGE gels and electrically transferred to PVDF membranes (Millipore, Burlington, MA, USA). The membranes were incubated with primary antibodies against Bcl-2 (1:1000, T40056, Abmart, Shanghai, China), Beclin1 (1:2000, ab207612, Abcam, Cambridge, UK), Bax (1:10,000, 50599-2-Ig, Proteintech, Wuhan, China), caspase3 (1:1000, T40044, Abmart, Shanghai, China), LC3B (1:2000, T40044, Abcam, Cambridge, UK), ZO-1(1:2000, 21773-1-AP, Proteintech, Wuhan, China), Occludin (1:10,000, 66378-1-Ig, Proteintech, Wuhan, China), β-actin (1:100,000, AC026, Abclonal, Wuhan, China), and GAPDH (1:10,000, 60004-1-Ig, Proteintech, Wuhan, China) at 4 °C overnight. They were then incubated in Peroxidase-Conjugated Goat Anti-Rabbit IgG(H+L) or Goat Anti-Mouse IgG(H+L) (Yeasen, Shanghai, China) diluted at 1:10,000 for 1 h at room temperature. After chemiluminescence detection had been performed using ECL reagents (Yeasen, Shanghai, China) and a Fusion Edge Multi-function Imaging System (Vilber, Collégien, France), the relative optical densities of the protein bands were analyzed using ImageJ software.

### 2.15. Co-Immunoprecipitation

Brain tissues were homogenized, and the centrifuged protein supernatants prepared for the co-immunoprecipitation assays. Protein A/G agarose (Beyotime, Shanghai, China) was firstly pre-washed with 1× PBS buffer, and then resuspended in the working solution containing antibodies against Beclin1 and Bcl-2. After being incubated for 15 min at room temperature, the mixture was placed on a magnetic rack to collect beads and antibodies. Next, the mixture of beads and antibodies was combined with the protein supernatants and left to rotate continuously at 4 °C overnight. This allowed the formation of the bead–antibody–antigen complex, which effectively absorbed the antigen. The complex of bead, antibody, and antigen was magnetically separated and subjected to five washes using 1× PBS buffer. Finally, the washed beads were resuspended in SDS-PAGE sample and used for protein immunoblot analysis.

### 2.16. Immunofluorescence Staining

Rats were anesthetized 24 h after surgery and perfused intracardially. Brains were fixed with 4% PFA and stored in 30% sucrose. After successful dehydration, the brain tissues were embedded in OCT compound for preservation. Serial coronal sections of 10–12 μm thickness were cut using a freezing cryostat (Leica, Wetzlar, Germany). After being washed three times with PBST, the sections were subsequently blocked with 0.3% Triton X-100 and 5% goat serum in PBS for 60 min at room temperature. After blocking, tissue sections were incubated overnight with the primary antibodies. The primary antibodies used were rabbit anti-ZO-1 (1:3000, 21773-1-AP, Proteintch, Wuhan, China), rabbit anti-Occludin (1:100, sc-133256, Santa Cruz, CA, USA), rabbit anti-LC3 (1:1000, ab192890, Abcam, Cambridge, UK), rabbit anti-Caspase3 (1:50, T40044, Abmart, Shanghai, China), and mouse anti-CD31 (1:100, MA1-80069, Invitrogen, Waltham, MA, USA). Afterwards, the sections were washed three times with PBST and then incubated with Alexa Fluor 647-conjugated goat anti-mouse secondary antibody (1:500, ab150115, Abcam, Cambridge, UK), as well as Alexa Fluo 488-conjugated goat anti-rabbit IgG antibody (1:500, ab150077, Abcam, Cambridge, UK). Finally, all sections were sealed with antifading mounting medium containing DAPI. Images were observed and analyzed using fluorescence microscopy (Leica Thunder, Wetzlar, Germany).

### 2.17. Statistical Analysis

The data were presented as the mean ± SD and were analyzed using GraphPad Prism software (version 8.0.1). The student’s *t*-test was used to assess disparities between two groups, whereas the one-way analysis of variance (ANOVA) was utilized to compare differences among three or more groups, followed by Tukey’s post-test involving multiple comparisons. Upon indication of unequal sample variances by the Brown–Forsythe test, the Brown–Forsythe ANOVA test was conducted, followed by the Games-Howell post-hoc test. Statistical significance was indicated when the value of *p* < 0.05.

## 3. Results

### 3.1. Acupuncture Extended the rt-PA Time Window to 6 h in the Embolic Stroke Model

**Figure 2:**
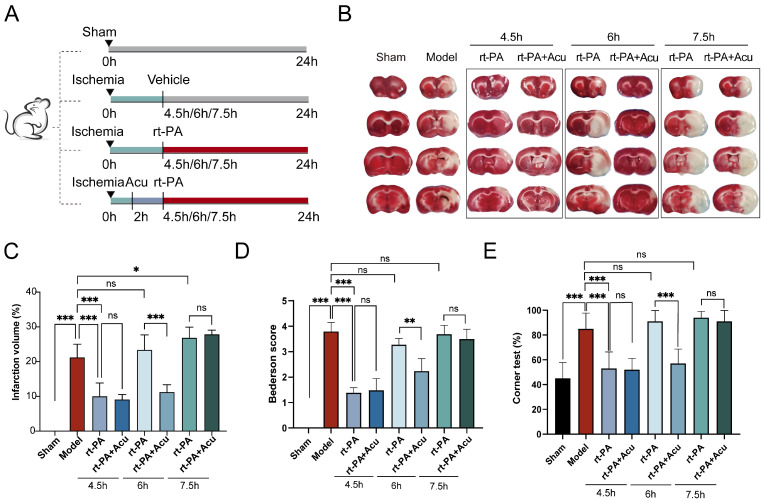
Acupuncture extends rt-PA time window to 6 h. (**A**) Schematic diagram of model establishment and rt-PA/acupuncture treatment. (**B**) Representative photographs of TTC-stained brain sections in each group. (**C**) Measurement of the size of the cerebral infarction determined by TTC staining (n = 4). (**D**) Bederson tests were performed at 24 h after the establishment of model and rt-PA treatment (n = 6). (**E**) Corner tests were performed to count the numbers of left and right turns at 24 h after the establishment of model and rt-PA treatment (n = 10). All data of results were presented as mean ± SD, * *p* < 0.05, ** *p* < 0.01, *** *p* < 0.001.

Evidence from clinical and experimental studies indicates that spontaneous clot dissolution and vascular recanalization can be observed within 1 h of clot injection, whereas maximum neurological deficit scores can be recorded at 2 h after ischemia [30,31,32]. Therefore, we performed acupuncture at the timepoint of 2 h after embolic stroke model established. To explore whether acupuncture intervention in advance could extend the rt-PA time window, apart from 4.5 h thrombolytic time, we conducted intravenous injection of rt-PA at several points beyond the time window (6 h, 7.5 h) in ischemic stroke (Figure 2A). We first used TTC staining to determine the extent of cerebral infarction. Figure 2B,C suggest that, compared to the model or rt-PA group, acupuncture combined with thrombolysis treatment within 6 h significantly decreased the percentage of cerebral infarction volume. However, at 7.5 h, rt-PA application or combination with acupuncture was ineffective for cerebral infarction.

Then we evaluated the effect of acupuncture on neurological score using the modified Bederson scale and Corner test at 24 h after operation. The results of the Bederson scores indicated that acupuncture significantly reduced neurological deficits induced by rt-PA thrombolysis within 6 h of stroke (Figure 2D). Moreover, in the Corner test, we found a higher left-turn preference when beyond the time window after right-side cerebral infarction. In the 6 h rt-PA group, acupuncture decreased the percentage of leftward turns. However, there was no statistically significant difference between the 7.5 h rt-PA group and the 7.5 h rt-PA combined with acupuncture group (Figure 2E). Taken together, these findings indicate that acupuncture could enable thrombolysis to play its part outside the time window and prolong the time window to 6 h.

### 3.2. Acupuncture Reduced Hemorrhagic Transformation and Cerebral Edema Caused by Delayed Thrombolysis with rt-PA

**Figure 3:**
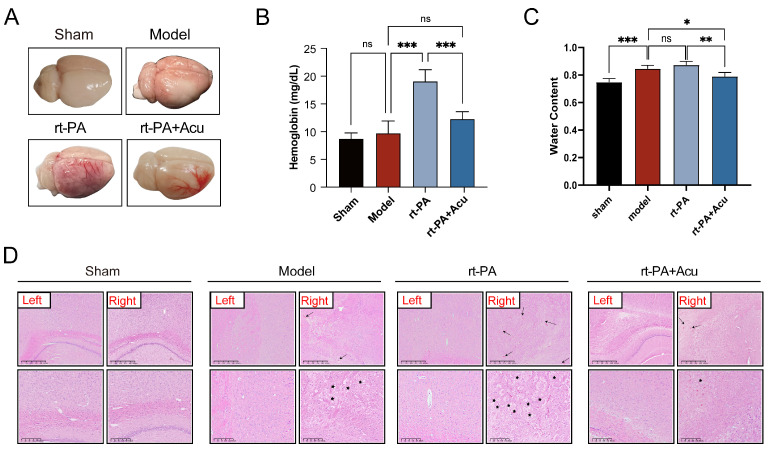
Effects of acupuncture in alleviating complications of thrombolysis. (**A**) Images of brain tissue on a bright field. (**B**) Hemoglobin levels in the ischemic hemisphere were examined at 24 h after the establishment of model and rt-PA treatment(n = 6). (**C**) Brain water content was examined at 24 h after the establishment of model and rt-PA treatment (n = 6). (**D**) Representative images of H&E staining in each group. Arrow indicates the location and amount of bleeding; asterisk indicates cell death and fragmentation. (scale bar = 625 μm for the images above, scale bar = 200 μm for the images below, n = 3). All data of results were presented as mean ± SD, * *p* < 0.05, ** *p* < 0.01, *** *p* < 0.001.

We further determine the role of acupuncture on delayed rt-PA thrombolysis at 6 h after stroke. Firstly, images of brain tissue revealed that intracranial hemorrhage possibly occurred with the right hemisphere lesion in those who received rt-PA outside the time window, and it can be prevented by acupuncture (Figure 3A). We used a spectrophotometric assay to measure the levels of hemoglobin in the ischemic hemisphere to assess hemorrhagic transformation. As shown in Figure 3B, rt-PA thrombolysis upregulated the content of hemoglobin in the supernatant of brain tissue homogenate, while acupuncture remarkably reversed hemoglobin level. Moreover, results of H&E staining showed the location and amount of bleeding, which are labeled by arrows (Figure 3D). We also saw cell death and fragmentation, marked by asterisks in the model and rt-PA images, which indicated that acupuncture administration notably mitigated these forms of tissue damage associated with ischemia and reperfusion (Figure 3D). In addition, brain water content was increased in the model and rt-PA groups. However, early treatment with acupuncture can decrease the brain water content (Figure 3C).

### 3.3. Transcriptome Analysis Revealed Acupuncture Can Ameliorate the Disruption of Blood Brain Barrier Components Caused by Delayed Thrombolysis

**Figure 4:**
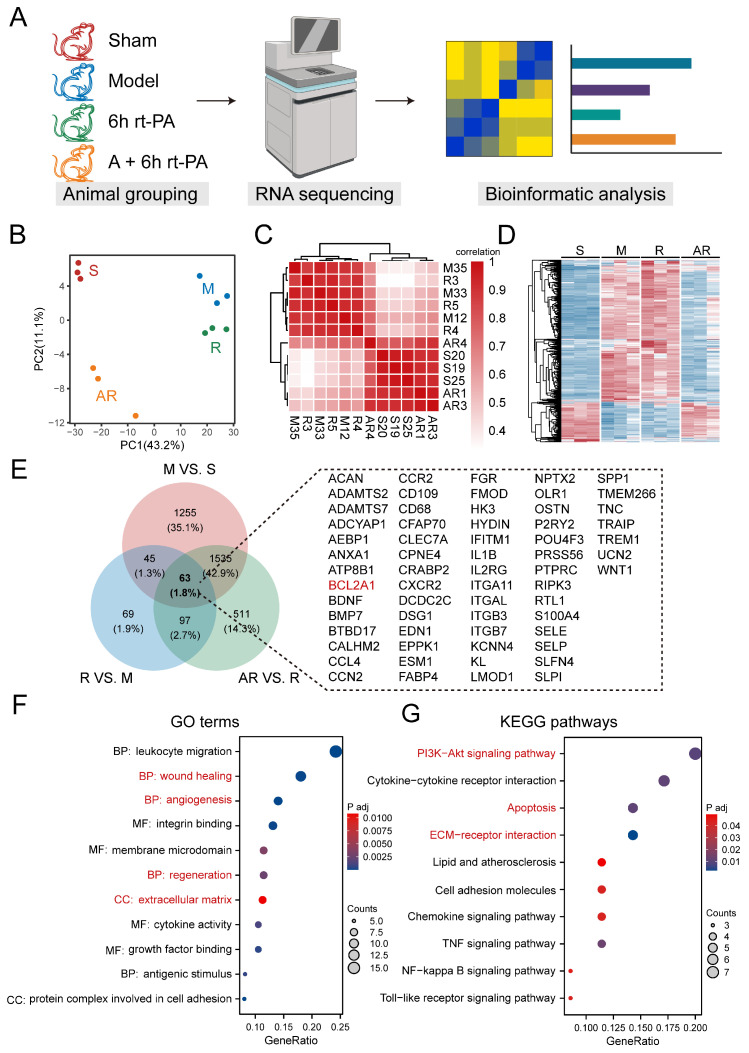
Transcriptome sequencing analysis of XNKQ acupuncture treatment for ischemia–reperfusion injury. (**A**) Schematic for animal grouping, RNA sequencing and transcriptomic analysis. (**B**) Principal component analysis (PCA) based on transcriptional profile among samples of different groups; each point represents one sample. (**C**) Spearman correlation was used to cluster samples and generate a heatmap. (**D**) The top 500 genes with the highest variance were selected for the heat map. (**E**) Venn diagram illustrating the intersection of DEGs. Specific gene information is presented in the panels to the right. (**F**,**G**) GO (**F**) and KEGG (**G**) analysis of the intersect DEGs. Pathways of interest are highlighted in red.

To identify the mechanisms by which acupuncture extends the thrombolysis window, transcriptome sequencing was conducted on cortical tissue after ischemic stroke. The schematic presentation of animal grouping for sequencing is indicated in Figure 4A. Principal component analysis revealed distinct gene expression clusters for each group. The sample distributions of the model and rt-PA thrombolysis group were clearly distinguished from the sham group, while a similarity between the sample of simultaneous administration of XNKQ acupuncture group and the sham group was observed (Figure 4B), in line with the transcriptome similarity (Figure 4C). We found that the tissue underwent lasting changes at the transcriptional level (Figure 4D). Differential expression analysis was performed to identify genes with significant expression changes. A total of 2898, 274, and 2206 DEGs were identified in the model, rt-PA thrombolysis, and acupuncture groups, respectively (Figures S2–S4, Table S1). We focused on the intersection of these DEGs, where potential targets of acupuncture intervention for extending the thrombolysis window may be identified. As a result, a total of 63 genes were identified in inter-group comparisons (Figure 4E). To explore the biological functions of these targets, we performed functional enrichment analysis. The results showed that biological processes, including inflammatory response, wound healing, and angiogenesis, are involved in the mechanisms by which acupuncture alleviates ischemia reperfusion injury in delayed thrombolysis (Figure 4F,G, Table S2). Extracellular matrix (ECM) production and vascular stability are important in maintaining the integrity of BBB, which suggested that the BBB could be a key target for acupuncture intervention [33]. The results are consistent with the theory reported previously that BBB dysfunction is an intrinsic driving factor for stroke incidence [34]. Additionally, it was observed that autophagy and apoptosis were both enriched, presenting an interesting phenomenon that will be described in later sections (Figure 4G). To sum up, transcriptome sequencing revealed that acupuncture ameliorated the disruption of blood brain barrier components caused by delayed thrombolysis.

**Figure 5:**
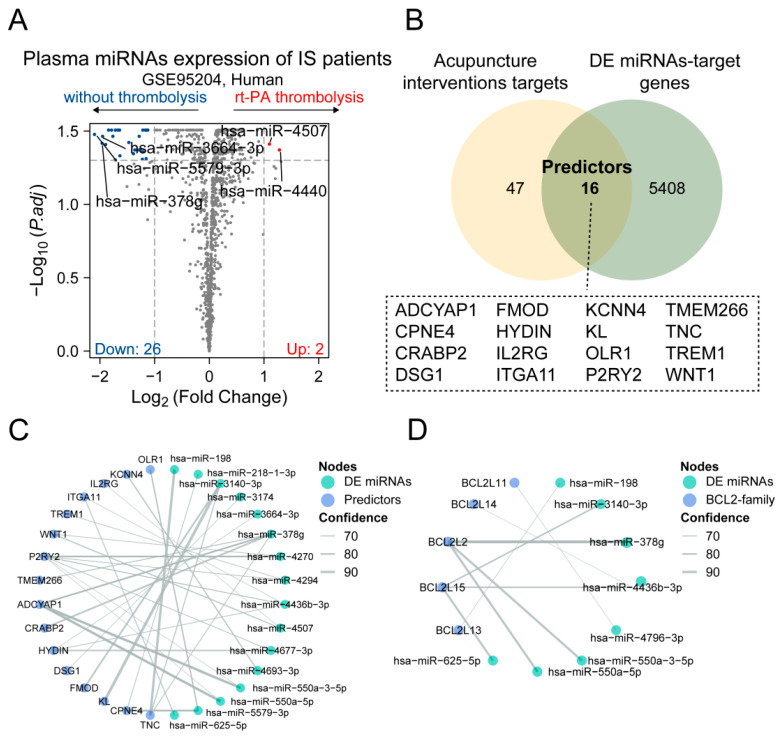
Identifying predictors of acupuncture treatment. (**A**) Volcano plot showing differentially expressed microRNAs (DE miRNAs) between ischemic stroke patients with and without thrombolysis in the public dataset. (**B**) The Venn diagram shows the number of overlapping acupuncture therapeutic targets in our study and DE miRNAs target genes predicted by the miRDB database. (**C**,**D**) The networks between DE miRNAs and 16 predictors (**C**) and the Bcl-2 family genes (**D**). Lines represent miRNA–target gene interactions and the thickness of the lines indicates confidence of target prediction.

To further identify predictors of acupuncture treatment, we accessed a public dataset that includes plasma miRNA sequencing data from ischemic stroke patients, distinguishing between those who received thrombolysis treatment and those who did not. We identified 28 differentially expressed micro-RNAs (DE-miRNAs) between patients (Figure 5A). Based on miRDB databases, we then sought potential target genes for the DE-miRNAs. By intersecting these targets with the acupuncture therapeutic targets identified in this study, we discovered a set of 16 predictors that are conserved across species (Figure 5B). The DE-miRNAs-target network displayed cooperative interactions between microRNAs, predictors, and the Bcl-2 family genes (Figure 5C,D). This intriguing finding suggests a potential overlap in the molecular mechanisms of thrombolysis treatment. In summary, we preliminarily revealed some potential predictors of acupuncture treatment, which could be pivotal for understanding the translatability of our findings from bench to bedside.

### 3.4. Acupuncture Suppressed Blood–Brain Barrier Leakage Induced by Delayed rt-PA Thrombolysis

**Figure 6:**
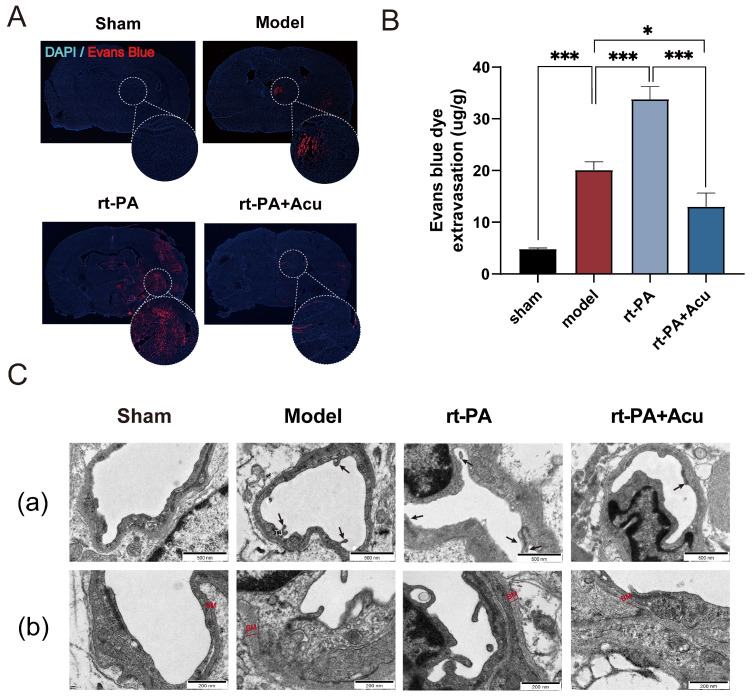
Acupuncture attenuated BBB damage induced by 6 h rt-PA thrombolysis. (**A**) Representative fluorescence images of brain tissue slices with EB staining. (**B**) EB dye leakage was examined at 24 h following modelling and rt-PA treatment. (**C**) Transmission electron microscopy of tight junctions (**a**, scale bar = 500 nm) and basement membrane (**b**, scale bar = 200 nm). Arrow indicates the position of TJ, (TJ, tight junction). Red straight line refers to the thickness of BM, (BM, basement membrane). All data of results were presented as mean ± SD, * *p* < 0.05, *** *p* < 0.001.

The occurrence of rt-PA thrombolysis complications is mainly related to the disruption of the BBB [35]. First, we evaluated BBB permeability by means of EB extravasation. Panoramic scanning of coronal brain slices showed that the red fluorescence signal of EB was remarkably aggregated in the cerebral cortex in stroke rats and that the fluorescence intensity was different between groups. The model group had increased fluorescence intensity and area, while delayed rt-PA administration induced a larger area of EB exudation out of the BBB. However, this alteration can be reversed in part by acupuncture (Figure 6A). At the same time, acupuncture significantly down-regulated a high amount of EB dye extravasation in brain tissue induced by delayed rt-PA thrombolysis and protected BBB integrity (Figure 6B). We further observed the structural changes of the BBB following cerebral infarction and thrombolysis under transmission electron microscopy. Images showed that the structure of tight junctions between microvascular endothelial cells were disrupted and gap clefts were seen in the model and rt-PA groups (Figure 6C), which ultimately lead to BBB leakage. However, this phenomenon was reversed by acupuncture enhancing tight junctions and reducing gaps between endothelial cells.

### 3.5. Acupuncture Restored the Down-Regulation Expression of Tight Junction Proteins

**Figure 7:**
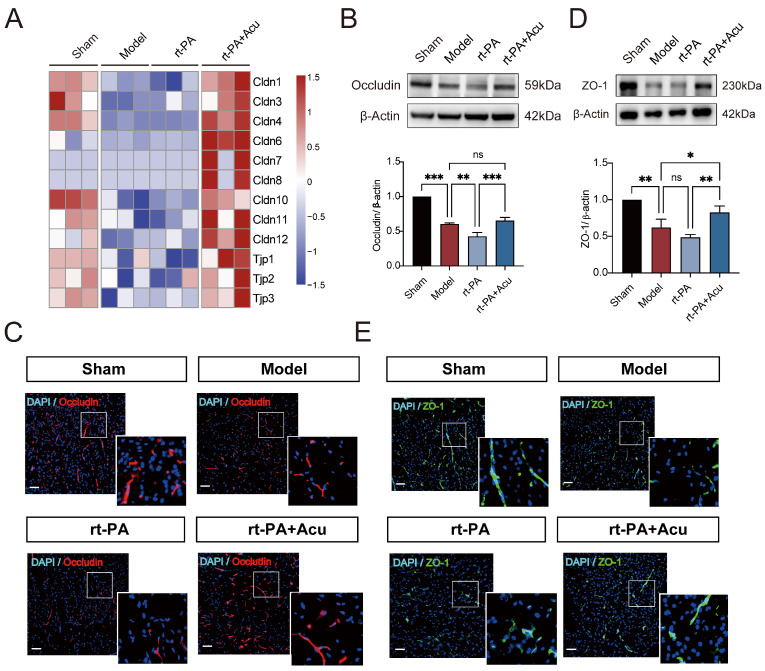
Acupuncture restored the down-regulation expression of Tight Junction Proteins. (**A**) Heatmap showing expression pattern of representative genes with tight junction. (**B**,**D**) Western blot analysis of Occludin (**B**) and ZO-1 (**D**) of 4 groups (Sham, Model, rt-PA and rt-PA+Acu, n = 3). (**C**,**E**) Representative immunofluorescence images of Occludin (**C**) and ZO-1 (**E**) in the ischemic penumbra area of 4 groups. (n = 3, scale bar = 50 μm). All data of results were presented as mean ± SD, * *p* < 0.05, ** *p* < 0.01, *** *p* < 0.001.

We further detected the expression of tight junction (TJ) proteins on vascular endothelial cells in order to verify the effect of acupuncture on BBB integrity. Transcriptome analysis revealed that acupuncture reversed the down-regulation of TJ genes (Figure 7A). Consistently, immunofluorescence staining and Western blot indicated that tight junctions including ZO-1 (Figure 7B,C) and Occludin (Figure 7D,E) were significantly down-regulated in the ischemic model. Delayed rt-PA thrombolysis further decreased the expression of ZO-1 and Occludin, while acupuncture significantly reversed the expression of these proteins.

### 3.6. Acupuncture Suppressed BMECs Apoptosis after Delayed rt-PA Thrombolysis

**Figure 8:**
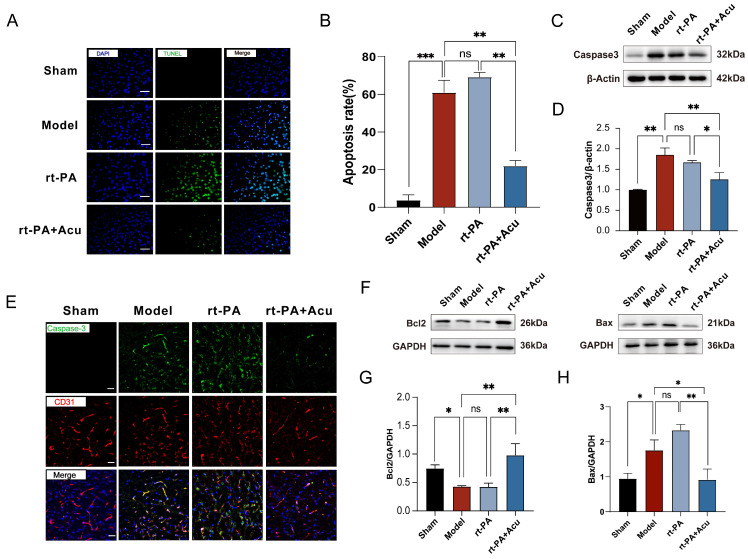
Acupuncture suppressed rt-PA thrombolysis-induced endothelial cell apoptosis. (**A**,**B**) Representative fluorescence images of TUNEL at 24 h after the establishment of model and rt-PA treatment (**A,** scale bar = 20 μm). Green TUNEL^+^ cells were used to assess the total apoptosis rates (**B**, n = 3). (**C**,**D**) Western blot analysis of Caspase-3 (n = 3). (**E**) Representative immunofluorescence images of Caspase-3 in endothelial cells (n = 3, scale bar = 20 μm). (**F**–**H**) Western blot analysis of Bcl-2 and Bax (n = 3). All data of results were presented as mean ± SD, * *p* < 0.05, ** *p* < 0.01, *** *p* < 0.001.

As the primary component of the blood–brain barrier, the survival of brain microvessel endothelial cells (BMECs) plays a critical role in maintaining the structural integrity of the BBB. It has been shown that apoptosis of BMECs, as the main form of cell death after stroke, thus leads to BBB damage [36]. To verify the occurrence of apoptosis, we first used TUNEL staining to detect apoptosis. There was a high percentage of TUNEL-positive cells in the Model and rt-PA groups, while acupuncture remarkably decreased the number of TUNEL-positive cells (Figure 8A,B).

To further confirm the effects of acupuncture on apoptosis, we then detected the protein expression level of several apoptosis markers, including Bax, Bcl-2, and Caspase-3. Expression of Caspase-3, a key executioner of apoptosis, was measured in the rat cerebral cortex. Compared to the model or rt-PA groups, acupuncture significantly decreased the protein level of Caspase-3 (Figure 8C–E). The B cell leukemia/lymphoma 2 (Bcl-2) family proteins regulate mitochondrial outer membrane permeability to control intrinsic apoptosis [37]. Similarly, acupuncture significantly increased the protein level of Bcl-2 and inhibited the protein expression of Bax. This change may be caused by an increase in Bcl-2 overexpression and decrease in Bax after acupuncture, which can control the release of Cyt-c and the activation of downstream Caspase-3 protease, mediating endothelial cell apoptosis.

### 3.7. Acupuncture Restored the Balance of BMECs Autophagy and Apoptosis through Modulation of Bcl-2-Beclin1 Complex

**Figure 9:**
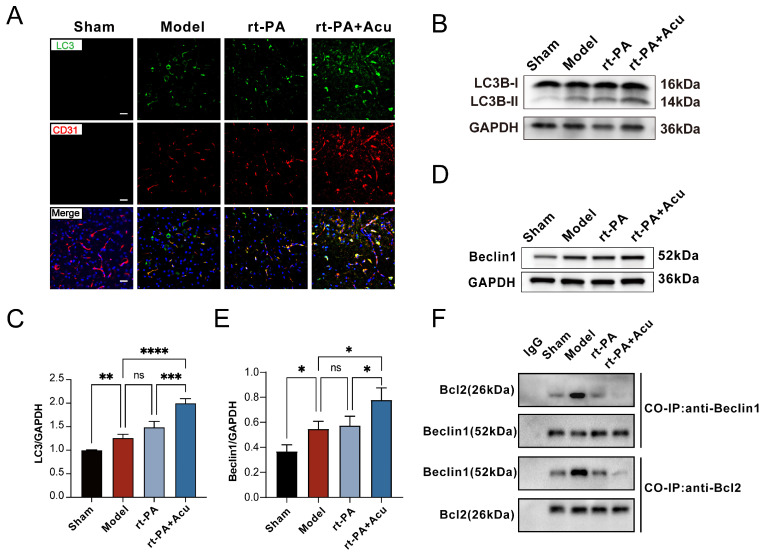
Acupuncture stimulates endothelial autophagy in blood vessels. (**A**) Representative immunofluorescence images of LC3 in endothelial cells (n = 3, scale bar = 20 μm). (**B**,**C**) Western blot (**B**) and quantitative analysis (**C**) of LC3 (n = 3). (**D**,**E**) Western blot (**D**) and quantitative analysis (**E**) of Beclin1 (n = 3). (**F**) Co-immunoprecipitation of Beclin1 and Bcl-2 binding of each group. All data of results were presented as mean ± SD, * *p* < 0.05, ** *p* < 0.01, *** *p* < 0.001, **** *p* < 0.0001.

An increasing number of studies have suggested that moderate autophagy activation could inhibit apoptosis to promote cell survival in the early stage of cerebral ischemia. Our pathway enrichment analysis showed that autophagy-related pathways were enriched (Figure 4G). Therefore, we wanted to further test whether autophagy was involved in the acupuncture’s ability to protect the BBB. We first used the double IF staining of LC3 and CD31, and the results revealed that acupuncture increased the co-localization of LC3-positive and CD31-positive cells compared to other groups, suggesting the formation of autophagosome in BMECs (Figure 9A). We then detected the protein expression level of LC3 and key regulatory proteins, including Beclin1 (Figure 9B–E). Consistently, acupuncture significantly increased the protein level described above. These results indicated that XNKQ acupuncture strongly activated autophagy of BMECs.

It has been documented that Bcl-2 regulates the balance between apoptosis and autophagy [38,39]. Normal conditions are characterized by a balance between autophagy and apoptosis, whereas in some diseases such as AIS this balance is disrupted [40]. Based on our findings that Bcl2a1 is target of acupuncture intervention (Figure 4E) and enrichment of both autophagy and apoptosis pathway at the same time (Figure 4G), it seems that autophagy and apoptosis processes undergo a regulation game of keep balance. We further investigated the balance between autophagy and apoptosis processes. Co-immunoprecipitation analyses of Bcl-2-Beclin1 complexes confirmed that Bcl-2 dissociated from Beclin1 after acupuncture intervention (Figure 9F). Based on the analysis of the two parts above, acupuncture was considered to suppress autophagy and induce apoptosis by regulating the Bcl-2/Beclin1interaction before rt-PA administration.

## 4. Discussion

In this study, we have demonstrated the effect and mechanism of XNKQ acupuncture on extending the rt-PA treatment time window and alleviating ischemia-reperfusion injury. The key findings are as follows: (1) Acupuncture alleviated infarction and neurological deficits and extended the thrombolysis window to 6 h. (2) The RNA-seq revealed 16 potential therapeutic predictors for acupuncture intervention, and the potential mechanisms were related to suppressing inflammation and restoring the function of the BBB and blood vessels. (3) Acupuncture suppressed BBB leakage and preserved tight junction protein expression. (4) Acupuncture prevents vascular endothelial damage and BBB disruption by modulating the interaction between autophagy and apoptosis. Moreover, acupuncture dissociated the Beclin1/Bcl-2 complex to activate autophagy and thereby alleviate apoptosis.

Performing rt-PA thrombolytic therapy in the early stage after cerebral infarction is currently the most effective method in clinical practice. In fact, less than 10% of patients with acute cerebral infarction have a chance to receive rt-PA thrombolysis treatment because of the strict time-window restrictions (3–4.5 h following stroke) and serious side effects. Seeking ways to improve the safety of rt-PA thrombolysis remains a challenge in clinical practice. Recently, a meta-analysis revealed that acupuncture, as a complementary and alternative medical therapy, combined with thrombolysis helps to improve the efficacy of thrombolysis. Moreover, our previous study confirmed that treatment with XNKQ acupuncture can relieve the adverse effects induced by rt-PA thrombolysis within the effective time window [41]. Shuigou (GV26) and Neiguan (PC6), the most significant acupoints of XNKQ acupuncture, are reported to promote the transporting of Qi and blood to the brain. In the theory of traditional Chinese medicine, accumulation of blood stasis, liver wind, and phlegm pool can obscure the brain, resulting in the closure of the orifices and concealment of the spirit, which ultimately lead to the attack of stroke. Regarding the Shuigou (GV26) acupoint, it is situated at the midpoint of the nasolabial groove, an area predominantly composed of sensory fibers under the innervation of the trigeminal nerve. Previous studies have shown that acupuncture stimulation at this point, particularly during reperfusion injury following a stroke, activates the specific somatosensory pathways of the head and face. This activation results in the release of calcitonin gene-related peptide (CGRP), suppression of neuropeptide Y (NPY) within the brain, and a decrease in the expression of angiotensin II and its type-1 receptor (AT1R) [42,43,44].These mechanisms collectively contribute to the amelioration of vascular dysregulation. Additionally, bilateral acupuncture at the Neiguan (PC6) is used to regulate blood flow and calm the spirit. Neiguan (PC6) is recognized as another of the principal points in the Xingnao Kaiqiao acupuncture prescription, which is known to significantly enhance cerebral blood flow and oxygen supply, thereby benefiting stroke patients. Studies have demonstrated that acupuncture at Neiguan improves cerebral blood flow by increasing the expression of proteins associated with angiogenesis, such as VEGF, bFGF, and CD34, as well as by restoring and improving the structure of endothelial cells and microvessels [45,46,47]. Our previous clinical findings indicate that Neiguan primarily enhances collateral circulation within the anterior cerebral circulation, which is a key aspect of improving blood supply in ischemic stroke patients [17].

The most important finding of our current study is that, by simultaneous administration of XNKQ acupuncture, the ideal time window of rt-PA thrombolysis could potentially be extended. The animal model used here is a rat embolic stroke model, which is similar to the pathological process of human ischemic stroke. Therefore, research conclusions based on this model have more reference significance. It has been shown here that XNKQ acupuncture extended the rt-PA time window to 6 h. Rats Rats treated with rt-PA and acupuncture, even 6 h after ischemic stroke, showed remarkable alleviation of brain ischemia and ischemia–reperfusion injury compared to those treated with rt-PA alone. The mechanisms behind these findings may include the following. Firstly, acupuncture modulates neuroinflammatory responses, which is essential in the initial treatment phase, influencing cytokine release to mitigate inflammation’s detrimental impact on neural tissue and enhance neuroprotection, with the most notable effects occurring within six hours of stroke onset [48,49]. Secondly, acupuncture activates neuroprotective pathways, including those involving BDNF and VEGF, which are vital for neuronal survival, neurogenesis, and the alleviation of neurological symptoms [50]. It also improves microcirculation in ischemic brain areas, facilitating the delivery of essential oxygen and nutrients to cells, thereby reducing ischemic damage [51,52]. Lastly, while the mechanisms are not completely understood, acupuncture appears to extend the therapeutic window for thrombolysis, potentially by protecting the blood–brain barrier and promoting angiogenesis, which supports better blood supply and drug delivery to the affected areas.

Interestingly, we observed that the protective effect of acupuncture at the 6-h point was not sustained at 7.5 h. While the exact mechanisms underlying this difference are not fully understood, several possibilities could be considered. On the one hand, the temporal dynamics of the ischemic cascade and subsequent reperfusion injury are complex, with various molecular and cellular processes unfolding over time [53,54]. It is possible that the initial protective mechanisms triggered by acupuncture, such as the upregulation of neurotrophic factors or the modulation of inflammatory responses, may have a limited duration of action or may be overwhelmed by the progressive pathological changes occurring at later stages [55]. On the other hand, the window of therapeutic opportunity for acupuncture may be narrower than previously thought, with the potential for diminishing returns as the ischemic injury progresses. The 6-h timeframe might represent a critical period where the brain is more responsive to acupuncture’s neuroprotective effects, and beyond this point, the treatment’s efficacy may decline due to the advanced stages of ischemic damage. Considering that our primary objective in this experiment was to investigate whether acupuncture could extend the therapeutic window of neuroprotection following ischemic stroke in rats, our future research will aim to dissect these mechanisms in more detail to optimize the timing and application of acupuncture in the treatment of ischemic stroke.

Ischemia–reperfusion injury involves a series of pathological responses. To gain a better understanding of the mechanisms involved in acupuncture intervention on the thrombolysis time-window extension and ischemia–reperfusion injury alleviation, we employed RNA-seq technology to obtain gene transcriptome profiles. A total of 63 potential therapeutic targets were identified, and enrichment analysis showed that, with the exception of suppressing inflammation, biological processes involved in restoring the function of BBB structure were wound healing and angiogenesis. In addition, to identify predictors of response to acupuncture treatment, we have discovered an intersection of 16 predictors across species. The roles of these predictors have been studied in the context of ischemic stroke. For instance, the WNT1 signaling pathway plays a crucial regulatory role in promoting the proliferation of endothelial cells within the central nervous system, maintaining the homeostasis of the blood–brain barrier, and synergizes with various angiogenic factors [56]. TREM1 is a receptor on myeloid cells that, upon binding with its ligand, can activate inflammatory responses. Research indicates that TREM1 may play a key role in the inflammatory response following cerebral infarction and could potentially serve as a biomarker for monitoring therapeutic outcomes and disease progression [57]. CRABP2 is involved in the metabolism and signaling of vitamin A, which has significant functions in the health and disease of the nervous system [58]. We believe that further investigation into these predictors could lead to the development of clinical translation, such as prediction-model construction and rapid biomarker screening, which would have significant implications for personalized medicine and therapeutic decision-making.

The BBB is a highly selective interface between the blood and brain parenchyma. The structure and integrity of blood vessels are crucial for the function of the BBB, while the extracellular matrix provides necessary support and regulation. The disruption of BBB structure and function was considered as one of the most direct restrictions in determining whether thrombolytic therapy could be performed. The role of inflammation pathways on BBB disruption and neuronal cell death after ischemia–reperfusion injury have been confirmed [59]. The integrity of the BBB was confirmed by transmission electron microscopy. We also measured EB leakage and brain water content, which reflected BBB disruption in the penumbra region. Experiments have demonstrated the improvement of vascular structure and vascular leakage by acupuncture. Additionally, BMECs are central to the barrier properties of the BBB, which have an apical domain and a basolateral domain, and are tightly bound with tight junctional (TJ) proteins [60]. TJs consist of cytoplasmic proteins (ZO-1) and transmembrane proteins (Occludin), and perform the function of closing the intercellular cleft, preventing free exchange between the CNS and vasculature [61]. The results showed that acupuncture treatment upregulated the expression of Occludin and ZO-1 after delayed rt-PA thrombolysis. This means cerebrovascular areas can better exert the barrier function and neuroprotective effects after acupuncture.

The disruption of the BBB by ischemia–reperfusion injury could cause BMECs damage, eventually leading to apoptosis [62]. Apoptosis is the predominant form of cellular demise following stroke. Under pathological conditions, accelerated apoptosis can cause neurodegenerative diseases or ischemic damage [63]. Bcl-2 and its family members are required for apoptosis through the intrinsic pathway. Bcl-2, together with Bax, modifies mitochondrial membrane potential and permeability, which releases regulatory proteins that activate cellular caspases, eventually the executor Caspase-3. BCL2A1, a member of the Bcl-2 family of proteins, was identified as one of the targets of XNKQ acupuncture intervention in our results. We found a striking decline of Bcl-2 and apparent activation of Bax and Caspase-3, accompanied by more TUNEL-positive cells and Caspase-3/CD31 double-positive cells after treatment with rt-PA alone, which contributed to BBB disruption. Importantly, this effect was reversed by acupuncture treatment, which indicates that acupuncture suppresses rt-PA thrombolysis-induced endothelial cell apoptosis.

In addition to the suppression of apoptosis, we also proposed the role of acupuncture in inducting autophagy. Both apoptosis and autophagy (PI3K/AKT) pathways were enriched in pathway analyses, which defined autophagy and apoptosis processes as the key pathways by which acupuncture protects BBB structure. Autophagy is the process by which cells transport damaged, denatured, or aging proteins and organelles to lysosomes for digestion and degradation, thereby avoiding the accumulation of redundant substances within the cell [64]. The PI3K/AKT pathway plays a significant role in regulating autophagy by affecting the mammalian target of rapamycin. We found that, compared with delayed rt-PA thrombolysis, acupuncture stimulated endothelial autophagy, which was confirmed by the formation of LC3 puncta, and up-regulation of Beclin-1 and LC3. Furthermore, we observed an intriguing phenomenon that the balance between autophagy and apoptosis pathways is challenged during ischemic stroke and rt-PA treatment. The results of immunoprecipitation indicated that acupuncture promotes the dissociation of Bcl-2 and Beclin1, thereby activating the autophagy process and inhibiting endothelial cell apoptosis, suggesting that the balance between autophagy and apoptosis may be a potential mechanism of acupuncture treatment in ischemic stroke.

Certainly, there are several limitations of this study. Firstly, we observed a beneficial effect of XNKQ acupuncture on improving post-thrombolysis vascular structural damage and vascular leakage. However, acupuncture showed multiple properties, such as the regulation of oxidative stress. Whether the protective effects of acupuncture on the BBB are due to other properties needs to be considered. Secondly, our study suggests that XNKQ acupuncture is involved in autophagy and apoptosis balance, but further work in vitro is needed to supplement these findings. Lastly, multiple cells such as neurons, microglia, astrocytes, and pericytes could be affected by XNKQ acupuncture after ischemic stroke. In future work, including single cell sequencing technology, we will focus on revealing heterogeneity that we cannot capture through global tissue expression profiles.

## 5. Conclusions

In conclusion, this study suggested XNKQ acupuncture as an adjuvant management for rt-PA thrombolysis to extend the time window and alleviate ischemia–reperfusion injury. We have shown here that XNKQ acupuncture extended the thrombolysis window by suppressing blood–brain barrier disruption and promoting the dissociation of Bcl-2 and Beclin1, consequently enhancing autophagy and inhibiting apoptosis. We hope that these findings provide a reliable theory basis for the clinical treatment of ischemic stroke with thrombolytic therapy within a broadened therapeutic window.

## Data Availability

The RNA-seq data produced in the study are uploaded and publicly available. The data can be found here: https://www.ncbi.nlm.nih.gov/geo/query/acc.cgi?acc=GSE262257 (accessed on 31 December 2023).

## Supplementary Materials

The following supporting information can be downloaded at https://www.mdpi.com/article/10.3390/brainsci14040399/s1, Figure S1: Cerebral blood flow was monitored by Laser speckle imaging during the surgery of embolic stroke model; Figure S2: Differential expression analysis and enrichment analysis result between Model and Sham groups; Figure S3: Differential expression analysis and enrichment analysis result between rt-PA thrombolysis and Model groups; Figure S4: Differential expression analysis and enrichment analysis result between simultaneous administration of XNKQ acupuncture group and rt-PA thrombolysis; Figure S5: The effect of acupuncture on the expression of tight junction protein; Table S1: The differential expression gene results in Sham, Model, rt-PA and Acu+rt-PA groups; Table S2: The enrichment analysis results of 63 potential therapeutic targets; Table S3: The differential expression miRNA and potential target genes predicted by miRNA database.
