# Supplementary material for: Acupuncture Extended the Thrombolysis Window by Suppressing Blood–Brain Barrier Disruption and Regulating Autophagy–Apoptosis Balance after Ischemic Stroke
Source: Brain Sci. 2024 Apr 19;14(4):399. doi: 10.3390/brainsci14040399 (PMC11048240; doi:10.3390/brainsci14040399)
Supplement: Supplementary file 1 [file brainsci-14-00399-s001.zip › Supplementary Figure.pdf]

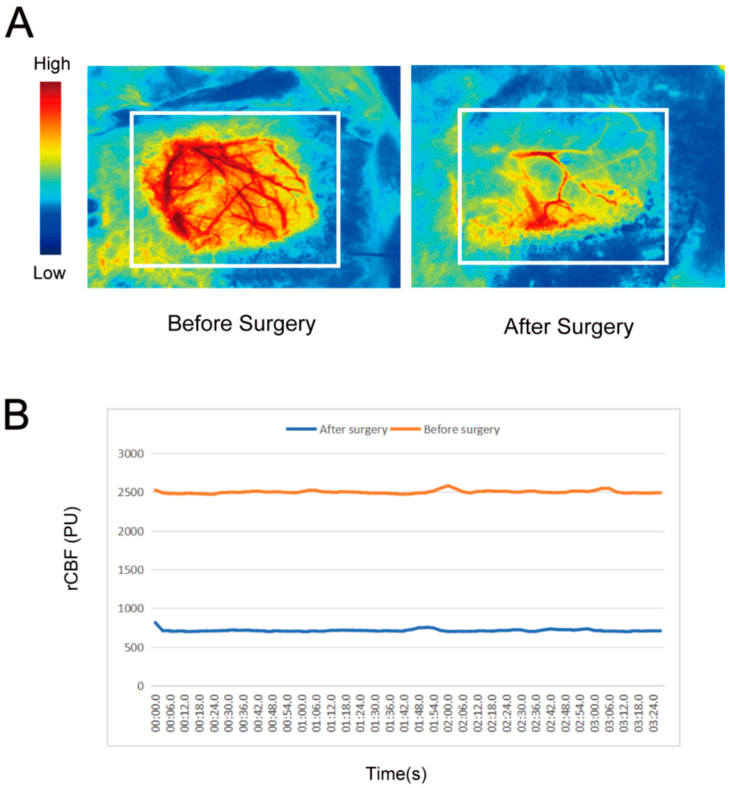

Figure S1. Cerebral blood flow was monitored by Laser speckle imaging during the surgery of embolic stroke model. A. Representative images of rCBF. White box is the place where we used to do the quantification. B. Compared to the rCBF before surgery, the reduction of 70% or more in rCBF was considered as successful induction of ischemia.

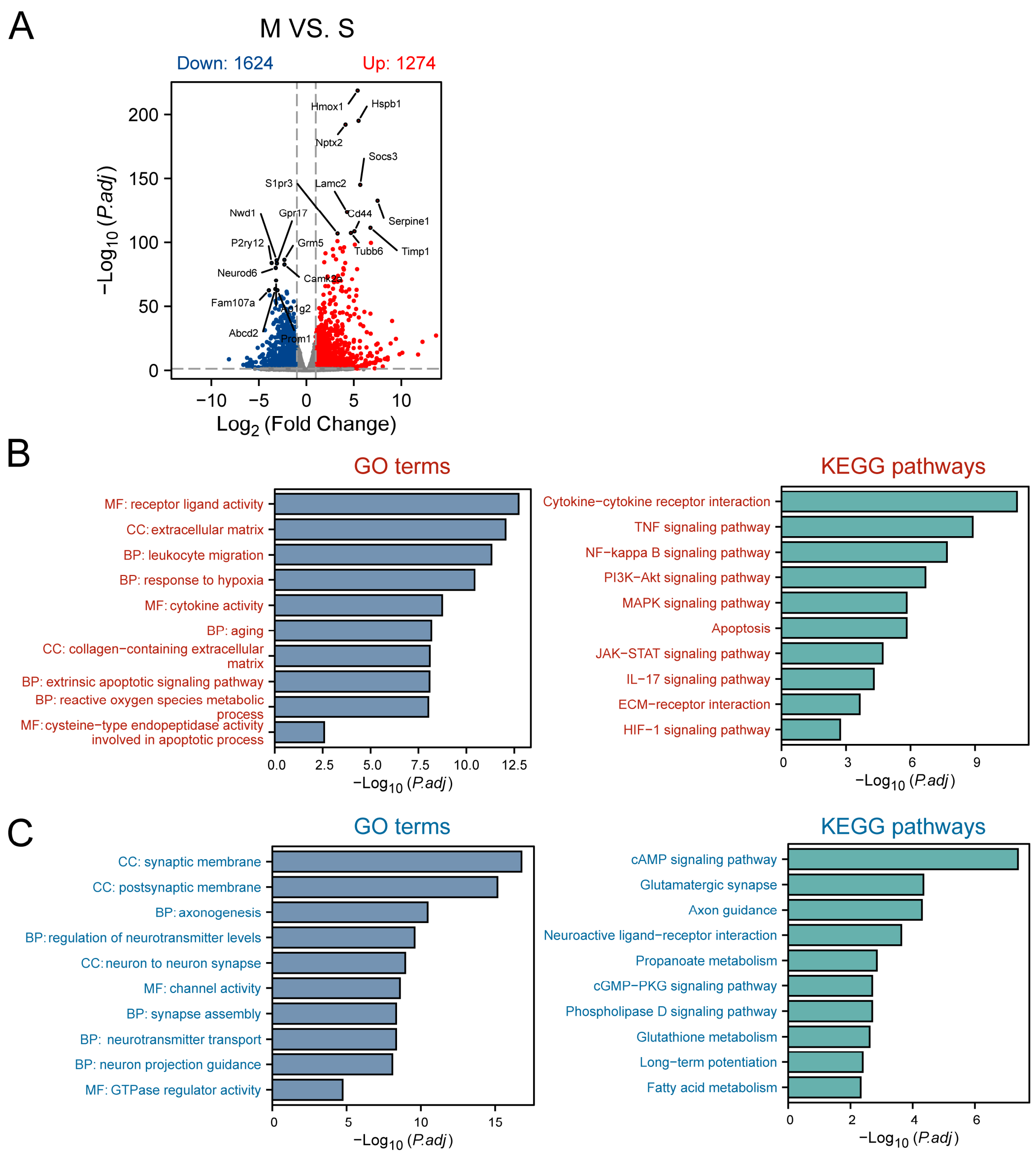

Figure S2. Differential expression analysis and enrichment analysis result between Model and Sham groups. (A) Volcano plot of differentially expressed genes. (B-C). Over-representation enrichment analysis of GO terms and KEGG pathways for up- (B) or down-regulated (C) genes

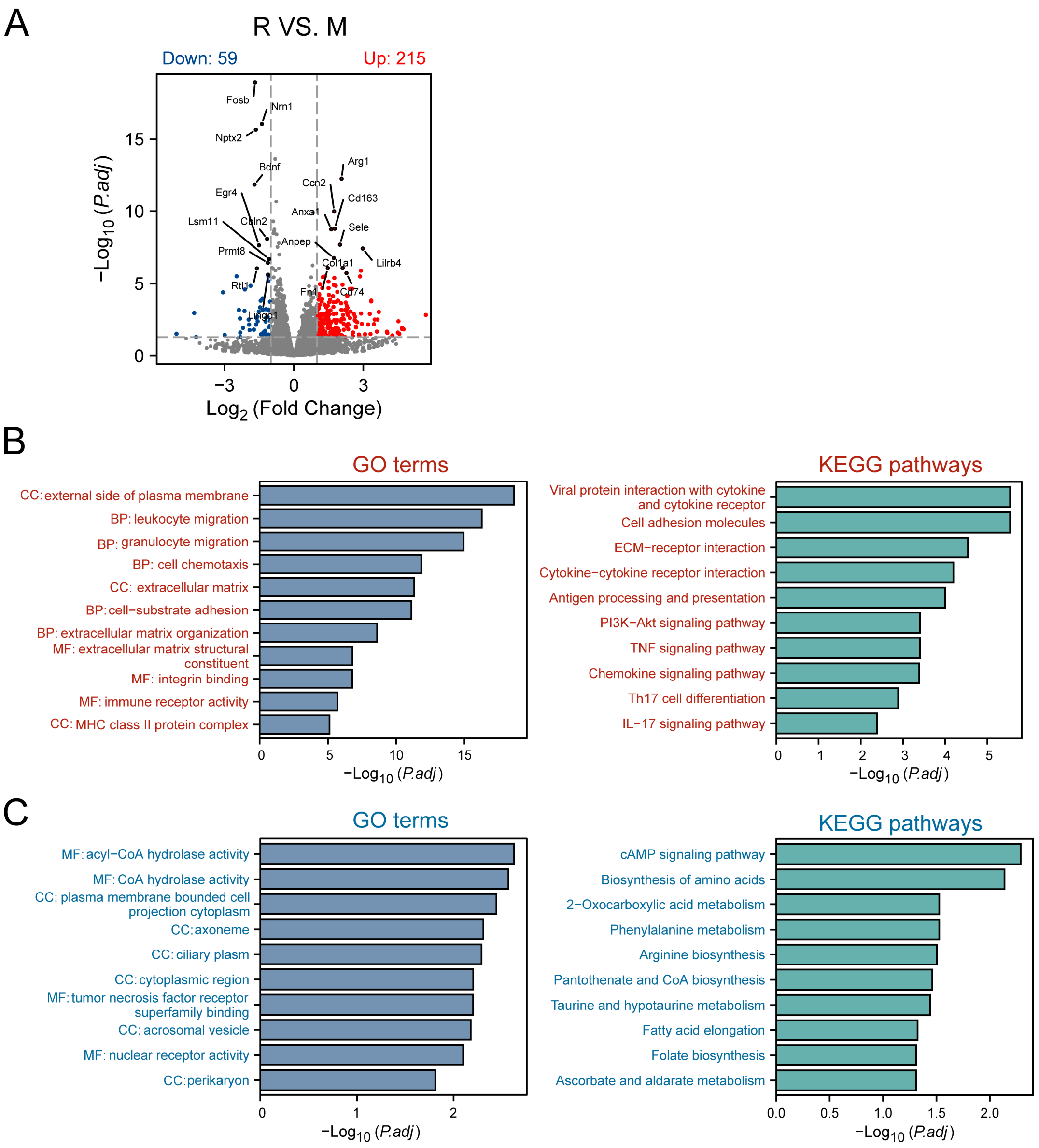

Figure S3. Differential expression analysis and enrichment analysis result between rt-PA thrombolysis and Model groups. (A) Volcano plot of differentially expressed genes. (B-C). Over-representation enrichment analysis of GO terms and KEGG pathways for up- (B) or down-regulated (C) genes

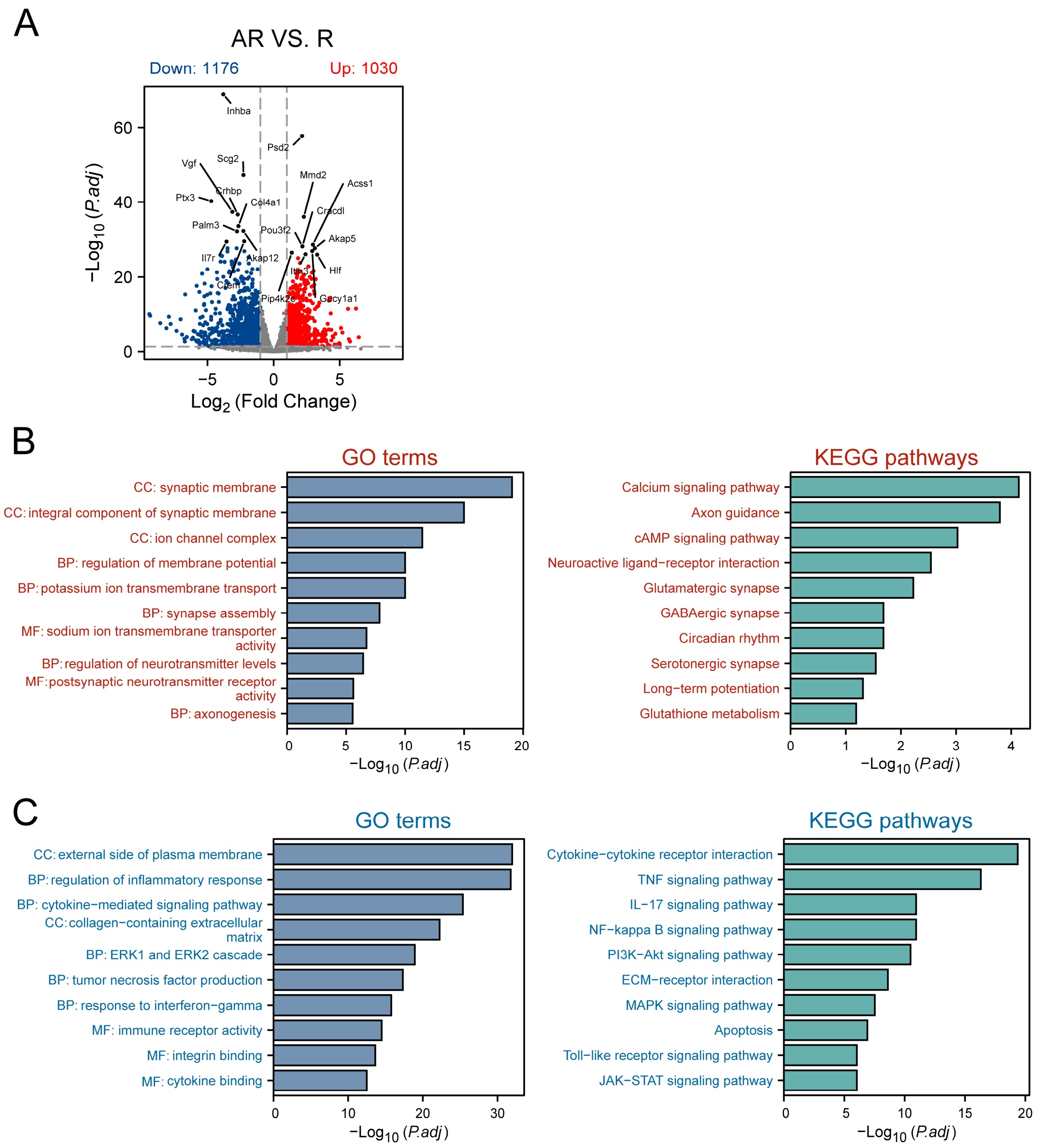

Figure S4. Differential expression analysis and enrichment analysis result between simultaneous administration of XNKQ acupuncture group and rt-PA thrombolysis. (A) Volcano plot of differentially expressed genes. (B-C). Over-representation enrichment analysis of GO terms and KEGG pathways for up- (B) or down-regulated (C) genes

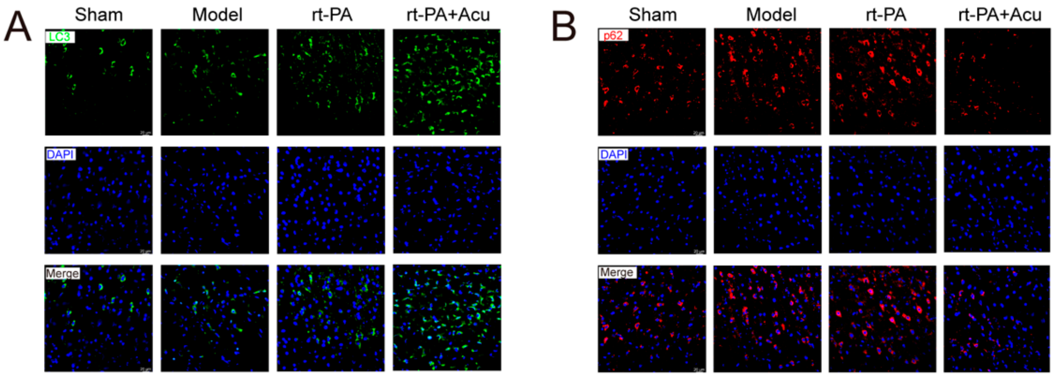

Figure S5. The effect of acupuncture on the expression of tight junction protein A. Representative images of LC3 immunofluorescence staining. B. Representative images of p62 immunofluorescence staining.
